# Supplementary material for: Co-Occurrence of L. monocytogenes with Other Bacterial Genera and Bacterial Diversity on Cleaned Conveyor Surfaces in a Swine Slaughterhouse
Source: Microorganisms. 2022 Mar 14;10(3):613. doi: 10.3390/microorganisms10030613 (PMC8948719; doi:10.3390/microorganisms10030613)
Supplement: Supplementary file 1 [file microorganisms-10-00613-s001.zip › supp_firgure S2.pdf]

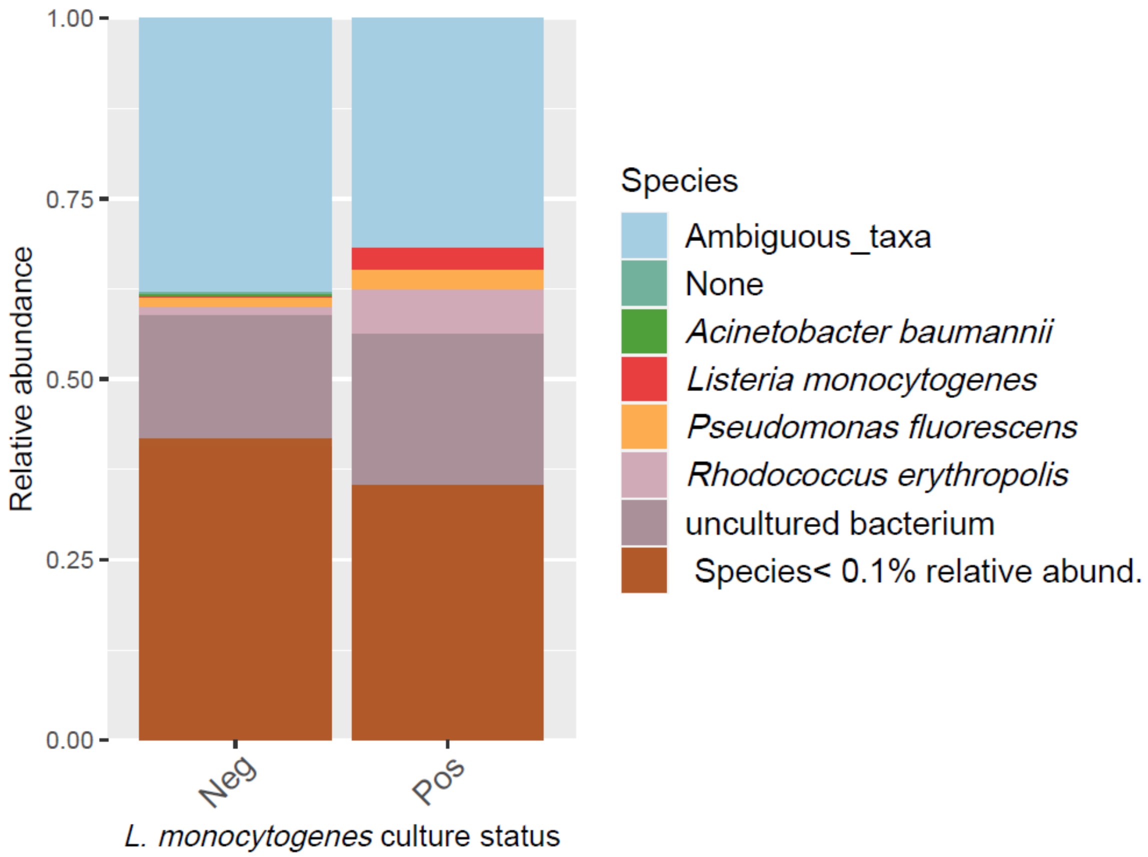

Supplementary Figure S2: Relative frequency of bacterial species identified from OTUs detected in cutting facility conveyor surfaces according to positive samples in *Listeria monocytogenes* culture-based method.
